# High protein and mRNA expression levels of TUBB3 (class III ß-tubulin) are associated with aggressive tumor features in esophageal adenocarcinomas

**DOI:** 10.18632/oncotarget.23112

**Published:** 2017-12-11

**Authors:** Heike Loeser, Simon Schallenberg, Moritz von Winterfeld, Lars Tharun, Hakan Alakus, Arnulf Hölscher, Elfriede Bollschweiler, Reinhard Buettner, Thomas Zander, Alexander Quaas

**Affiliations:** ^1^ Institute of Pathology, University of Cologne, Cologne, Germany; ^2^ Department I of Internal Medicine, Center for Integrated Oncology (CIO), University of Cologne, Cologne, Germany; ^3^ Department of General, Visceral and Cancer Surgery, University of Cologne, Cologne, Germany; ^4^ Department of Thorax and Oesophageal Surgery, Agaplesion Markus Krankenhaus, Frankfurt/Main, Germany

**Keywords:** esophageal adenocarcinoma, TUBB3, RNA-in-situ-hybridization, immunohistochemistry

## Abstract

**Background:**

Esophageal adenocarcinomas show an increasing incidence in the Western world and their overall survival remains low. Microtubules are multifunctional cytoskeletal proteins involved in crucial cellular roles, including maintenance of cell shape, intracellular transport, meiosis, and mitosis. Microtubulus-TUBB3 was found overexpressed in several carcinomas suggesting a significant role in cancer development. High levels of TUBB3 expression were also described to be associated with poor clinical outcome in various cancers. It was shown that overexpression of TUBB3 could be related to reduced efficiency of taxane-based targeting anticancer drugs in several cancer types.

**Results:**

There is a statistically significant association between high TUBB3 protein and TUBB3 mRNA expression and shortened survival (p<0,0001). Prognostic impact of TUBB3 expression is seen in patients with and without multimodal treatment. Multivariate analysis revealed a strong TUBB3 expression to be an independent prognosis factor. Validation of protein expression by mRNA in situ hybridization underlines the credibility of the immunohistochemical results.

**Discussion:**

Our study emphasized the significant importance of TUBB3 in esophageal adenocarcinoma. TUBB3 serves as an independent prognostic marker and may be a valuable biomarker for routine application in esophageal adenocarcinoma especially to address the need for adjuvant treatment in individuals following neoadjuvant therapy and surgery. Future prospective studies are needed which include the results of TUBB3 in preoperative biopsy material to proof the prognostic impact of TUBB3.

**Materials and Methods:**

280 esophageal adenocarcinomas that underwent primary surgical resection or resection after neoadjuvant therapy were analyzed by mRNA-in-situ-hybridization (RNAscope^®^) and by immunohistochemistry (TUBB3 rabbit monoclonal antibody; Epitomics).

## INTRODUCTION

Esophageal cancer is the eighth most common malignant tumor diagnosed in the world. Although squamous cell cancer is the most frequent tumor type, esophageal adenocarcinomas show an increasing incidence in the Western world [1]. Despite improvements in perioperative treatments, the overall survival of patients with esophageal carcinoma remains low. Preoperative radiochemotherapy or chemotherapy alone is evidence based therapeutic tool for many esophageal cancer patients, although just few reliable markers exist to predict response to the therapy. Biomarkers to evaluate a prognostic stratification for the effect of neoadjuvant radiochemotherapy and to predict the need for an adjuvant treatment are urgently needed.

Microtubules are multifunctional fibrous cytoskeletal proteins involved in crucial cellular roles, including maintenance of cell shape, intracellular transport, meiosis, and mitosis. Microtubules are composed of polymers of α- and β-tubulin heterodimers, existing as multiple isotypes with a complex pattern of distribution among different tissues [2]. Class III β-tubulin (βIII-tubulin; alias TUBB3) is a microtubule protein, normally expressed in cells of neuronal origin [3]. It is thought that the microtubulus-TUBB3 isotype is responsible for generating the highly dynamic microtubules required for neurite formation and motility in neuronal tissues [4]. It was found overexpressed in several solid tumors, including non-small cell lung cancer [5], ovarian cancer [6, 7], urothelial carcinoma of the bladder [8] and head and neck squamous cell carcinoma [9], suggesting a significant role in cancer development [6]. High levels of βIII-tubulin expression were also described to be associated with poor clinical outcome in various cancers, including non-small cell lung cancer, ovarian cancer, urothelial carcinoma of the bladder, as well as prostate cancer [2, 3, 7, 10]. Overexpression of βIII-tubulin was shown to be related to reduced efficiency of taxane-based targeting anticancer drugs in several cancer types [11]. In a study of gastric cancer, pre-treatment immunohistochemical evaluation of βIII-tubulin was predictive for taxane-based chemotherapy in advanced tumor stages [12]. However, genetic alterations of βIII-tubulin are rare in gastric cancer [13] and esophageal adenocarcinoma (TCGA data, provisional). Immunohistochemical TUBB3 status reveals differences between gastric and esophageal adenocarcinoma in a small cohort of 126 and 106 tumor samples respectively [14]. In this study we analyzed the protein and mRNA-expression of βIII-tubulin in a cohort of 280 esophageal adenocarcinomas.

## RESULTS

### Immunohistochemistry

A total of 280 patient with esophageal adenocarcinomas that underwent transthoracic esophagectomy were immuno-histochemically interpretable via TMA analysis. Reasons for noninformative cases (51 spots; 18.2%) included lack of tissue samples or absence of unequivocal cancer tissue in the TMA spot.

**Figure 1:**
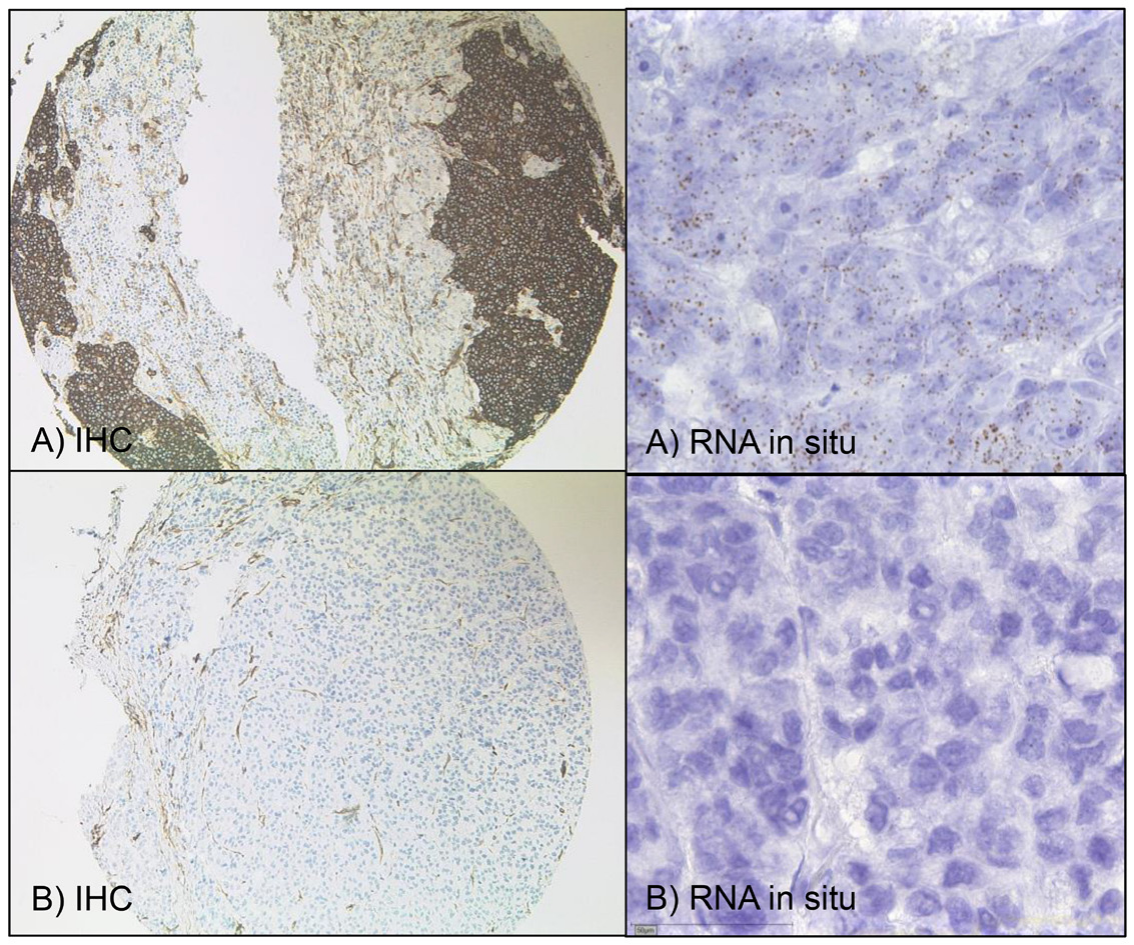
TUBB3 – immunohistochemistry and RNA-in-situ **(A)** Immunohistochemically (IHC, x100) strong TUBB3 positivity and same tumor with high level TUBB3-mRNA with more than 15 dots per cell (x400) expression. **(B)** TUBB3 is negative in this tumor (IHC, x100) and no/or just less than one signal per 10 cells of mRNA expression in the same tumor (x400).

**Figure 2:**
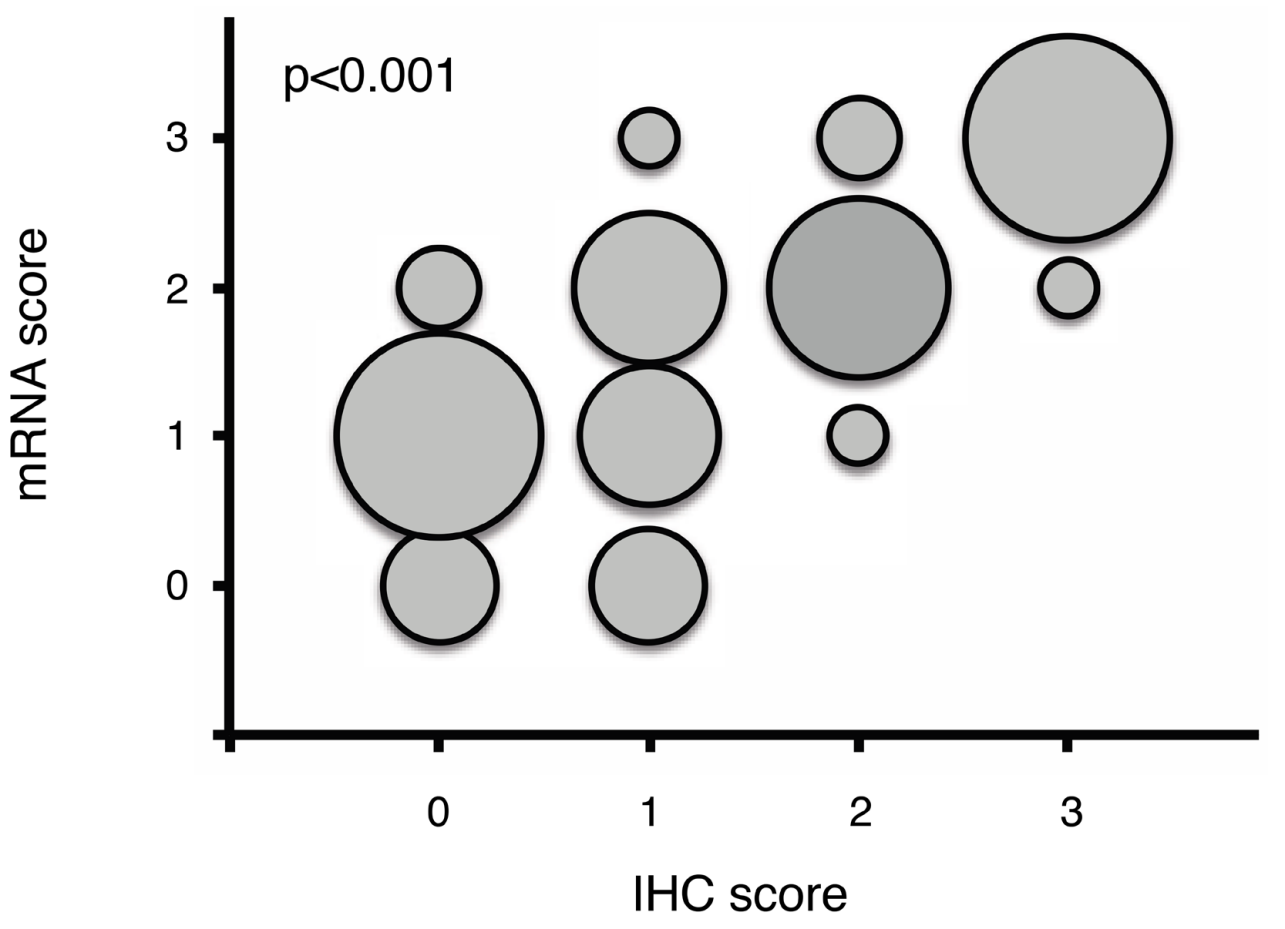
Correlation of mRNA and protein (immunohistochemical) results for TUBB3 The size of the circles shows the co-occurrence of mRNA and protein. The results demonstrate an excellent correlation of mRNA expression and protein measured by immunohistochemistry (p<0,001).

TUBB3 immunostaining was localized to the cytoplasm of the cells. In total 77% of the 280 adenocarcinomas revealed a positive immunostaining, from which 81 (29%) carcinomas were weakly positive (Score 1), 70 (25%) showed a moderate staining for βIII-tubulin (Score 2) and 64 (23%) adenocarcinomas were strongly positive (Score 3) according to the criteria mentioned above (Figures 1, 2).

### mRNA-*in situ* single cell expression analysis

A total of 280 adenocarcinomas were available for measuring the TUBB3 mRNA in the same TMA format using RNA-Scope technology (Figure 1). We found an excellent correlation of mRNA expression and protein levels measured by immunohistochemistry for TUBB3 – tumors exhibited high protein levels showed elevated mRNA levels and vice versa (interrater agreement 0,88 (95% CI 0,85-0,92).

### TUBB3 expression correlates with advanced tumor stages

For all 280 patients with IHC and mRNA data we observed a higher expression of TUBB3 in more advanced tumors (Table 1)

**Table 1A:** Patient’s characteristics and correlation TUBB3 immunohistochemistry

Factor		Negative		Weak/moderate		Strong		Sign.
		n	%	n	%	n	%	p
**Total**	n=280	65	23%	151	54%	64	21%	--
**Gender**								0.812
Male	n=253	59	23%	135	53%	59	23%	
Female	n= 27	6	22%	16	59%	5	19%	
**Age**								0.283
< 50y	n= 45	15	33%	18	40%	12	27%	
50-70y	n=164	33	20%	94	57%	37	23%	
> 70y	n= 71	17	24%	39	55%	15	21%	
**cT or pT-cat**								0.003
pT1/2	n= 52	19	37%	31	60%	2	4%	p-trend=
c/pT3/4	n=228	46	20%	120	53%	62	27%	0.0001
**pN or ypN-cat**								0.037
pN0	n=108	29	27%	63	58%	16	15%	p-trend=
pN+	n=172	36	21%	88	51%	48	28%	0.023

**Table 1B:** Patient’s characteristics and correlation TUBB3 mRNA

Factor		Negative		Weak/moderate		Strong		Sign.
		n	%	n	%	n	%	p
**Total**	n=280	75	27%	145	52%	60	21%	--
**Gender**								0.449
Male	n=253	70	28%	128	50%	55	22%	
Female	n= 27	5	19%	17	63%	5	19%	
**Age**								0.178
< 50 y	n= 45	15	33%	20	45%	10	22%	
50 −70y	n=164	35	21%	92	56%	37	23%	
>70y	n= 71	25	35%	33	47%	13	25%	
**cT or pT-cat**								0.0015
pT1/2	n= 52	23	44%	25	48%	4	8%	p-trend=
c/pT3/4	n=228	52	23%	120	52%	56	25%	0.0003
**pN or ypN-cat**								0.016
pN0	n=108	35	32%	59	55%	14	13%	p-trend=
pN+	n=172	40	23%	86	50%	46	27%	0.005

Also for patients without neoadjuvant therapy we found a significant trend for more TUBB3 expression in advanced pT-categories (pT3/4) compared to pT1/2 (p-trend=0.0001) and in patients with pN+ compared to patients without lymph node metastasis (p-trend=0.023).

Enhanced TUBB3 expression (Score 3) is significantly associated with an advanced depth of tumor infiltration (p<0.003) and the existence of lymph node metastases (p=0.023). For patients with advanced tumor infiltration the kind of therapy – primary surgery vs neoadjuvant therapy, or chemoradiation vs. chemotherapy had no influence on the TUBB3 expression (Table 1).

### TUBB3 expression is associated with patient survival

**Figure 3:**
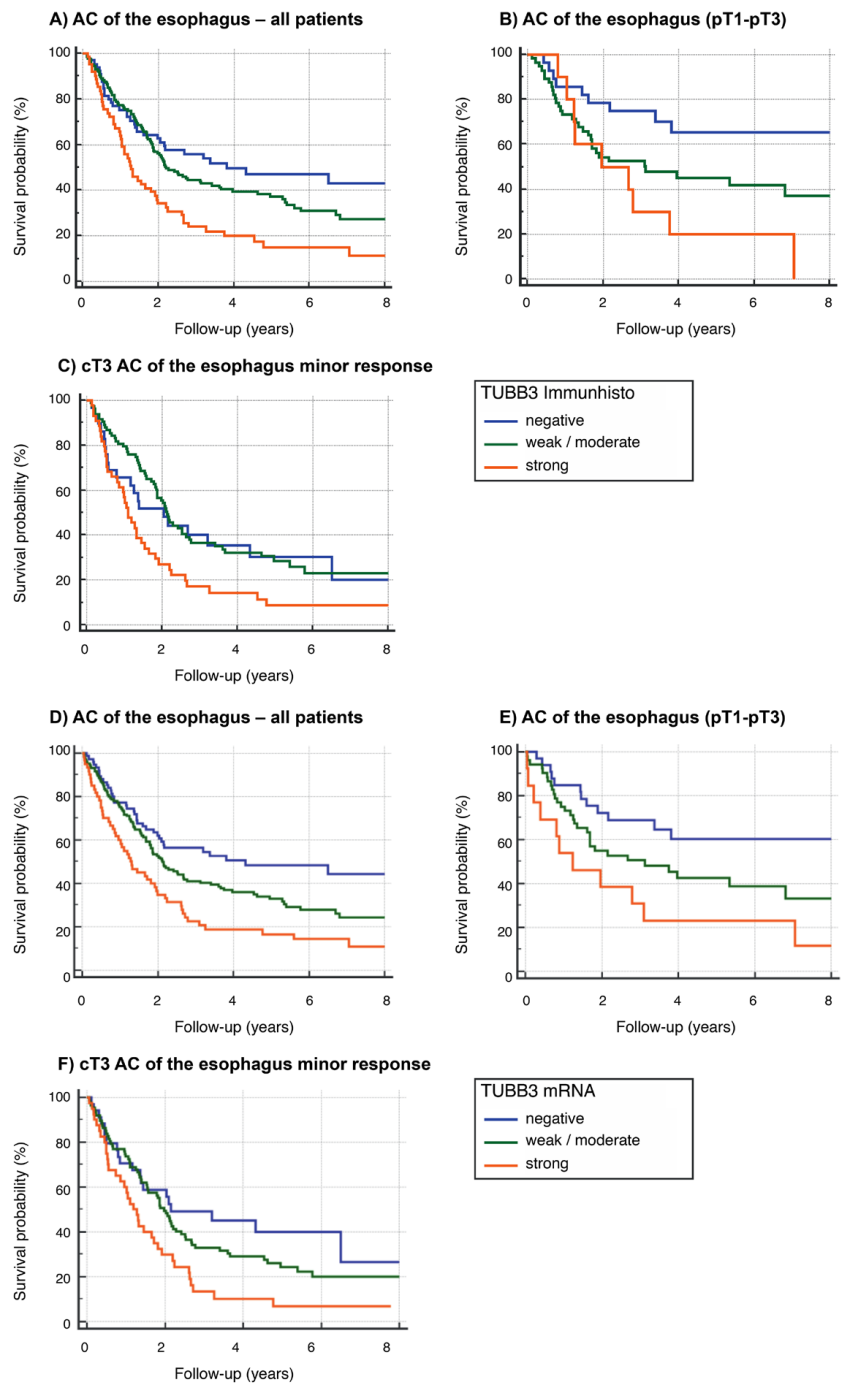
Survival data **(A-C)** TUBB3 in the cohort of all patients (n=280) using protein expression analysis: 16% 5-year survival rate in the group of high-level (strong) TUBB3 expression. Strong TUBB3 expression is correlated with shortened survival (p=0,0003). (B) TUBB3 in the group of patients without neoadjuvant treatment (surgery, only) (n=98): TUBB3 is correlated with advanced post-surgery tumor stage (p=0,0071). (C) Tumors with advanced local tumor stage according to clinical evaluation (cT3) and minor response to neoadjuvant treatment (n=160) (definition of minor response: more than 10% vital tumor cells. Minor response measured by pathologist at surgery specimen). High-levels of TUBB3 expression is correlated with shortened survival in the group of minor responders (p=0,0050). **(D-F)** TUBB3 in the cohort of all patients (n=280) using mRNA expression analysis: 17% 5-year survival rate in the group of high-level (strong) TUBB3 expression. Strong TUBB3 expression is correlated with shortened survival (p=0,0002). (E) TUBB3 in the group of patients without neoadjuvant treatment (surgery, only) (n=98): TUBB3 is correlated with advanced post-surgery tumor stage (p=0,0304). (F) Tumors with advanced local tumor stage according to clinical evaluation (cT3) and minor response to neoadjuvant treatment (n=161) (definition of minor response: more than 10% vital tumor cells. Minor response measured by pathologist at surgery specimen). High-levels of TUBB3 expression is correlated with shortened survival in the group of minor responders (p=0,0055).

The 5-year survival rate of the whole cohort was 35% and the median overall survival 2.15 (95% CI=1.86-2.67) years. The TUBB3 protein high expressing patients demonstrated a significantly (p=0.0003) worse prognosis with 16% 5-year survival rate and a median overall survival of 1.27 (95% CI=1.05-1.92) years compared to the low expressing patients (5 year survival 46%, median overall survival 3,82 years. (Table 2A, Figure 3A and 3B). Highly, similar results were observed for high mRNA expressing patients (p=0.0002) (Table 2B). This association is true for patients with primary surgery (protein p=0.0071, mRNA p=0.0304), after neoadjuvant radiochemotherapy (protein p=0.0130, mRNA 0.0305) and with minor response to the neoadjuvant treatment (protein p=0.0050, mRNA 0.0055) (Table 2A and 2B). Especially in patients with minor response to neoadjuvant treatment we thought to evaluate the prognostic value of TUBB3 expression as the majority of high risk patients with locally advanced disease are within this group. We observed again a significant association of TUBB3 protein and mRNA expression in this group (protein p=0.0050, mRNA 0.0055). In this group multivariate analysis including age, gender, TUBB3 expression and lymph node metastases revealed a strong TUBB3 protein expression (p=0.044) and ypN+ (p=0.003) to be independent prognostic factors for patients with minor response after neoadjuvant therapy (Figure 3C; Table 3A). Similar results were obtained for mRNA expression (p=0.021) (Figure 3F; Table 3B).

**Table 2A:** Univariate analysis of prognosis TUBB3 immunohistochemistry

Factor TUBB3	n	5 y SR	Hazard-ratio	Median survival	Signif.
			(95% CI)	(95% CI)	
**All patients**	**280**	35%		2.15 (1.9-14.0) y	p=0.0003
negative	65	49%	Reference	3.82 (2.0-14.0) y	
weak/moderate	151	36%	1.27 (0.9-1.8)	2.21 (1.9-3.6) y	
strong	64	16%	2.21 (1.4-3.5)	1.27 (1.0-1.9) y	
**Primary surgery**	**98**	46%		3.76 (1.9-10.1) y	p=0.0071
negative	28	65%	Reference	13.21 (3.8-14.0) y	
weak/moderate	58	43%	1.94 (1.1-3.6)	3.10 (1.6-10.0) y	
strong	12	17%	3.60 (1.4-9.3)	1.24 (1.0-2.8) y	
**neoadjuvant**	**182**	27%		1.92 (1.5-2.2) y	p=0.0130
negative	37	36%	Reference	2.04 (1.8-6.5) y	
weak/moderate	93	32%	1.02 (0.7-1.6)	2.16 (1.9-2.8) y	
strong	52	13%	1.74 (1.0-2.9)	1.10 (0.9-1.7) y	
**neoadjuvant**					
**minor response**	**160**	23%		1.85 (1.4-2.2) y	p=0.0050
negative	30	33%	Reference	2.04 (0.8-6.5) y	
weak/moderate	85	28%	1.01 (0.6-1.6)	2.12 (1.8-2.5) y	
strong	45	9%	1.87 (1.1-3.3)	1.10 (0.7-1.5) y	

**Table 2B:** Univariate analysis of prognosis for TUBB3 mRNA

FactorTUBB3	n	5 y SR	Hazard-ratio	Median survival	Signif.
			(95% CI)	(95% CI)	
**All patients**	**280**	33%		2.09 (1.8-2.6) y	p=0.0002
negative	75	48%	Reference	4.32 (2.1-14.0) y	
weak/moderate	145	33%	1.46 (1.1-2.0)	2.13 (1.8-2.7) y	
strong	60	17%	2.32 (1.5-3.6)	1.27 (0.9-1.9) y	
**Primary surgery**	**98**	46%		3.37 (1.9-10.0) y	p=0.0304
negative	33	60%	Reference	13.21 (3.4-14.0) y	
weak/moderate	52	42%	1.91 (1.1-3.4)	3.12 (1.7-6.8) y	
strong	13	23%	3.13 (1.2-8.5)	1.23 (0.4-3.1) y	
**neoadjuvant**	**182**	25%		1.88 (1.6-2.2) y	p=0.0305
negative	42	39%	Reference	2.09 (1.4-6.5) y	
weak/moderate	93	28%	1.22 (0.8-1.8)	2.07 (1.8-2.5) y	
strong	47	15%	1.84 (1.1-3.0)	1.32 (1.0-1.9) y	
**neoadjuvant**					
**minor response**	**161**	23%		1.85 (1.4-2.2) y	p=0.0055
negative	34	40%	Reference	2.16 (1.4-6.5) y	
weak/moderate	87	24%	1.33 (0.9-2.1)	1.97 (1.5-2.4) y	
strong	40	7%	2.23 (1.3-3.8)	1.19 (0.9-1.7) y	

**Table 3A:** Cox-regression analysis for patients with minor response

Factor	n =160	Hazard ratio	95% CI	p
**TUBB3 Immunhisto**				
Negative	30	1 (Reference)	-	-
Weak/moderate	85	1,006	0.60-1.67	0.979
Strong	45	1,758	1.01-3.05	0.044
**ypN-category**				
ypN0	55	1 (Reference)	-	
ypN+	105	1,843	1.22-2.77	0.003
**Age**				
< 50 years	28	1 (Reference)	-	-
50-70 years	103	1,093	0.67-1.78	0.721
>70 years	29	1,277	0.69-2.36	0.436
**Gender**				
male	140	1 (Reference)	-	
female	20	0.847	0.48-1.48	0.564

**Table 3B:** Cox-regression analysis for patients with minor response

Factor	n =161	Hazard ratio	95% CI	p
**TUBB3 mRNA**				
Negative	34	1 (Reference)	-	-
Weak/moderate	87	1,296	0.78-2.14	0.311
Strong	40	1,946	1.11-3.42	0.021
**ypN**-category				
ypN0	55	1 (Reference)	-	
ypN+	106	1,841	1.20-2.74	0.005
**Age**				
< 50 years	28	1 (Reference)	-	-
50-70 years	104	0.976	0.60-1.58	0.921
>70 years	29	1,164	0.63-2.13	0.623
**Gender**				
male	141	1 (Reference)	-	
female	20	0.826	0.47-1.44	0.504

## DISCUSSION

**Figure 4:**
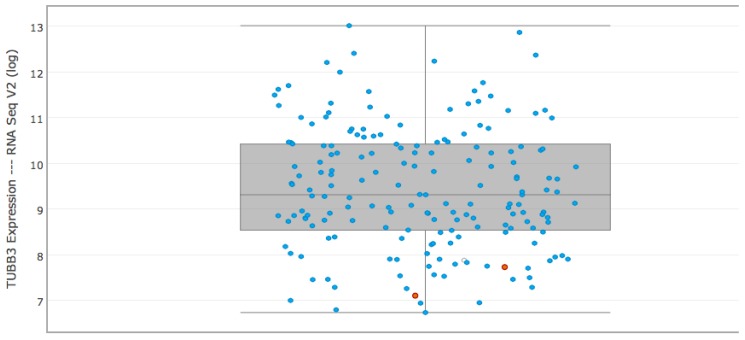
RNA expression of TUBB3 according to the results of TCGA consortium in esophageal carcinomas The graph above derived from cbioportal (www.cbioportal.org) according to the results of TCGA expression data in esophageal carcinoma. These results can serve as a kind of independent control cohort.

The results of our study demonstrate that the mRNA- and corresponding immunohistochemical TUBB3 protein expression in adenocarcinomas of the esophagus serve as a prognostic marker. High TUBB3 expression is associated with adverse prognosis, including advanced tumor stage, lymph node metastasis and minor response to neoadjuvant therapy. Furthermore TUBB3 expression is significantly associated with shortened survival. Our multivariate analysis revealed TUBB3 as an independent prognostic factor in the group of minor responders when jointly analyzed with lymph node metastasis. The expression results of the TCGA-esophageal carcinoma consortium can serve as a kind of an independent control cohort. According to their RNA sequencing data TUBB3 expression is a common finding in esophageal adenocarcinoma with a wide range of expressions levels (up to 13x log RNA expression; compare Figure 4) and confirm our results with more than 75% of tumors showing a TUBB3 expression of which are 25% highly TUBB3 overexpressed. To our best knowledge just one other study investigate the TUBB3 expression in esophageal adenocarcinoma using immunohistochemistry, only [14]. This study investigated 106 esophageal adenocarcinomas and was able to detect TUBB3 protein in 32,1% of these tumors. This discrepancy to our result is probably due to the different antibodies used. Our data were fully congruent with the results of earlier studies investigating TUBB3 expression in other carcinomas (breast, prostate, renal tumors, stomach, e.g.) [10, 14–16]. In gastric cancer, high-level expression of TUBB3 is associated with poor response to taxane-based chemotherapy and a significantly shorter progression-free-survival [12], in prostate adenocarcinomas TUBB3 was shown to be an independent prognostic marker showing a strong link with early PSA recurrence independent of grade and stage [10]. A previous study in breast carcinoma reported that TUBB3 messenger RNA expression was associated with reduced survival, although the authors did not identify a significant association when TUBB3 expression was determined by TUBB3 IHC analysis [15].

The associations described above between high TUBB3 expression levels and unfavorable tumor features are probably linked to TUBB3s role in preserve the plasticity of microtubules, invasive growth or cell motility. Increased plasticity of microtubules offer improvements to tumor cells due to the fact that migration/invasion require dis-/assembly of microtubules. Miura et. al. focused on biomarkers with potential (in-) sensitivity to (chemo)-therapeutic agents like taxane and emphasized that a carcinoma-cell based TUBB3 expression predicts the insensitivity to a taxane-based therapy most likely by its ability to keep microtubules in a more dynamic state or influence the drug-binding options ([17–19]). According to the results of the Magic trail and in consensus to the German S3-guidelines the esophageal adenocarcinoma of our cohort underwent preoperative chemotherapy also got taxane-containing drugs. About 60% of our patients demonstrate with poor response to this treatment (so called minor-responder) [20–22]. Our TMA tumor cohort revealed an even higher content of minor responders (64%) mainly due to the fact of missing analyzable tumor rests in major responders, forbidding a detailed analysis on the association between TUBBIII expression and response. According to the results of the present study, especially the subgroup of minor responders demonstrated with worse prognosis in association to high TUBB3 protein levels. These data raise the possibility that TUBB3 represents a biomarker with a potential clinical utility. This is further supported by the fact that our approach of analyzing features on a TMA specimen, measuring 1.2 mm in diameter, closely models the analysis of small biopsy specimens, in which comparable amounts of tissue are available. Our results suggest that TUBB3 may be a valuable prognostic marker for routine application in adenocarcinoma of the esophagus, especially to address the need for adjuvant treatment in individuals following neoadjuvant therapy and surgery. Future prospective studies are needed which include the results of TUBB3 in preoperative biopsy material to proof the prognostic impact of TUBB3.

## MATERIALS AND METHODS

### Patients and tumor samples

We analyzed formalin-fixed and paraffin embedded material of 280 from total 691 patients with esophageal adenocarcinomas that underwent primary surgical resection or resection after neoadjuvant therapy between 1999-2012 at the Department of General, Visceral and Cancer Surgery, University of Cologne, Germany. Standard surgical procedure was laparotomic or laparoscopic gastrolysis and right transthoracic en bloc esophagectomy including two-field lymphadenectomy of mediastinal and abdominal lymph nodes. Reconstruction was performed by high intrathoracic esophagogastrostomy as described previously [23]. Patients with advanced esophageal cancer (cT3, cNx, M0) received preoperative chemoradiation (5-FU, cisplatin, 40Gy as treated in the area prior the CROSS trial) or chemotherapy. Follow-up data were available for all patients. Patient characteristics are given in Table 1. Depending on the effect of neoadjuvant chemo- or radiochemotherapy there is a preponderance of minor responders, defined as histopathological residual tumor of ≥10% [24].

For Tissue Microarrays (TMA) one tissue core from each tumor was punched out and transferred into a TMA recipient block. TMA construction was performed as previously described [25, 26]. In brief, tissue cylinders with a diameter of 1.2 mm each were punched from selected tumor tissue blocks using a self-constructed semi-automated precision instrument and embedded in empty recipient paraffin blocks.

Four μm sections of the resulting TMA blocks were transferred to an adhesive coated slide system (Instrumedics Inc., Hackensack, NJ) for mRNA-in-situ and immuno-histochemistry.

### Immunohistochemistry

Immunohistochemistry (IHC) was performed on TMA slides using the primary antibody specific for ßIII-tubulin (rabbit monoclonal antibody, dilution 1:500; Epi- tomics Inc., Burlingame, CA) with a *Bond Max automated system* (Leica). Nerves served as an internal control.

The TUBB3 staining intensity was scored manual by two pathologists (A.Q. and H.L.) according to a 4-tier-scoring system. We defined Score 3+ as a strong staining of ≥30% of tumor cells or moderate staining ≥70%. Score 2+ was defined as weak staining in >70% or moderate staining in >30 and ≤70% or as strong staining in ≤30% of tumor cells. Score 1+ was assigned when ≤70% of tumor cells were weakly positive or ≤30% were moderately stained. Less staining was defined as negative (Score 0). Discrepant results were resolved by consensus review.

### RNA-*in-situ* (RNA-Scope)

The RNAscope assay was performed according to manufacturer’s instruction [27].

In brief, paraffin-embedded TMA blocks were cut in 5 μm sections, pretreated according to extended protocol (30 minutes for pretreatment 2 and 3), digested and hybridized at 40°C in the HybEZ oven with human TUBB3 mRNA probe provided by Advanced Cell Diagnostics Europe. Incubation time with Hematoxylin was 10 seconds.

Target expression was compared to both negative (dapB) and positive (PPIB) controls. Scoring of signals was done as recommend by the manufacturer with no staining or less than one molecule per 10 cells = score 0, 1-3 dots/cell = score 1, 4-9 dots/cell = score 2, 10-15 dots/cell = score 3 and >15 dots/cell = score 4. DapB score was 0 and PPIB score was 2. Positivity was defined as a score >0.

### Statistical analysis

Clinical data were collected prospectively according to a standardised protocol. Chi-square statistics were calculated for factor frequencies with a significance level of p<0.05.

Prognosis was calculated including all types of mortality beginning at the date of surgery. Univariate analysis of prognosis used Kaplan-Meier plots to describe survival distribution and the log-rank test to evaluate survival differences. Five year survival rate, the median survival with its 95% confidence interval (C.I.) and the Hazard-Ratio with its 95% C.I. for each factor levels were calculated. The multivariate analysis of survival used Cox regression analysis to identify independent prognostic variables. The level of significance was set to p<0.05.

Statistical analyses were carried out using the statistic program SPSS for Windows version 22.0. For graphic presentation of the results, MedCalc Statistical Software version 16.8.4 (MedCalc Software bvba, Ostend, Belgium; https://www.medcalc.org; 2016) was used.
